# Performance Analysis of Resonantly Driven Piezoelectric Sensors Operating in Amplitude Mode and Phase Mode

**DOI:** 10.3390/s23041899

**Published:** 2023-02-08

**Authors:** Phillip Durdaut, Michael Höft

**Affiliations:** Chair of Microwave Engineering, Department of Electrical and Information Engineering, Faculty of Engineering, Kiel University, Kaiserstr. 2, 24143 Kiel, Germany

**Keywords:** AM, amplitude noise, cantilever, detectivity, limit of detection, magnetic field sensor, magnetoelastic delta-E effect, phase noise, PM, resonator, sensitivity

## Abstract

Piezoelectric layers coupled to micromechanical resonators serve as the basis for sensors to detect a variety of different physical quantities. In contrast to passive sensors, actively operated sensors exploit a detuning of the resonance frequency caused by the signal to be measured. To detect the time-varying resonance frequency, the piezoelectric resonator is resonantly excited by a voltage, with this signal being modulated in both amplitude and phase by the signal to be measured. At the same time, the sensor signal is impaired by amplitude noise and phase noise caused by sensor-intrinsic noise sources that limit the reachable detectivities. This leads to the question of the optimum excitation frequency and the optimum readout type for such sensors. In this article, based on the fundamental properties of micromechanical resonators, a detailed analysis of the performance of piezoelectric resonators in amplitude mode and phase mode is presented. In particular, the sensitivities, the noise behavior, and the resulting limits of detection (LOD) are considered and analytical expressions are derived. For the first time, not only the influence of a static measurand is analyzed, but also the dynamic operation, i.e., physical quantities to be detected that quickly change over time. Accordingly, frequency-dependent limits of detection can be derived in the form of amplitude spectral densities. It is shown that the low-frequency LOD in phase mode is always about 6 dB better than the LOD in amplitude mode. In addition, the bandwidth, in terms of detectivity, is generally significantly larger in phase mode and never worse compared with the amplitude mode.

## 1. Introduction

Micromechanical cantilevers are among the simplest micromechanical resonators and are utilized to detect a variety of physical quantities. Generally, a microcantilever’s surface can adsorb molecules that generate surface stress where the adsorption of specific molecules can be achieved by coating one surface with a thin layer of a material that shows an affinity to certain molecules in the environment [1]. Instead of that adsorption mechanism, a functional layer can also be utilized to introduce surface stress (e.g., by utilizing a magnetostrictive layer) by way of nonmolecular quantities such as, e.g., magnetic fields [2]. The induced surface stress, in turn, affects the resonance frequency of the micromechanical resonator [3], the evaluation of which allows conclusions to be drawn about the respective physical quantity being measured (measurand). Among many other applications, resonant cantilever sensors for the detection of, e.g., humidity [3], heat [4], biomolecules [5,6], volatile organic compounds [7], explosives [8], magnetic fields [9,10,11,12,13,14], and atomic forces (atomic force microscopy, AFM) [15,16] have been presented.

Regardless of the physical quantity to be detected, the essential task of such measuring systems is to reconstruct the measurand by continuously detecting changes in the resonance frequency. One possibility, widely used in AFM, is to excite the cantilever in the vicinity of its resonance frequency utilizing a piezoelectric actuator while detecting the mechanical motion of the cantilever’s tip using a laser beam and a photosensitive detector ([17] p. 196). Concerning the signal-to-noise ratio (SNR), the optical beam deflection method is equivalent to the Michelson interferometer [18]. However, to eliminate the optical displacement sensor that occupies most of the space in such systems, piezoresistive [19], capacitive [20], and piezoelectric [21] readout schemes have been presented [22].

**Figure 1:**
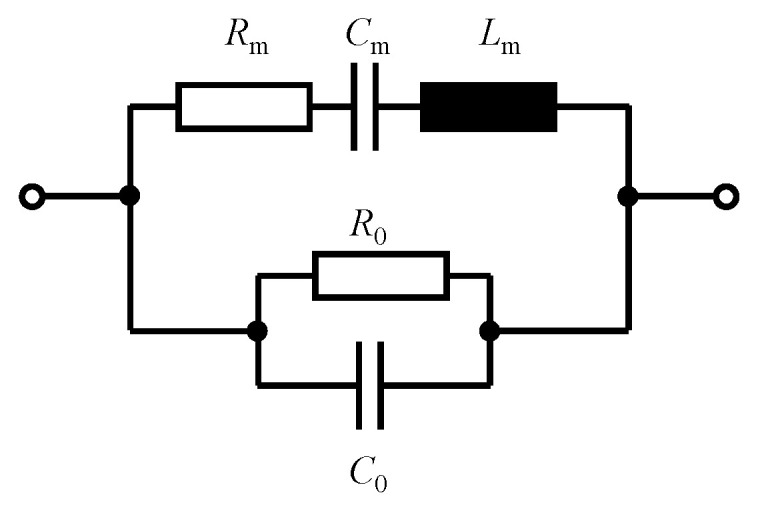
Lumped-element equivalent electrical circuit of a piezoelectric resonator.

Micromechanical cantilevers combined with a piezoelectric layer offer the great advantage that the piezoelectric material can be used to drive the resonator, as well as to detect its state. Although more sophisticated structures have been presented [23], most piezoelectric cantilevers have two electrodes, so the cantilever can be interpreted as a simple electrical two-pole (one-port) with a lumped-element equivalent electrical circuit in accordance with Figure 1. Based on the analogies between mechanical and electromechanical systems [24], the electromechanical resonator can be modeled as an electrical series resonance circuit with the motional resistance $R_{m}$, the motional capacitance $C_{m}$, and the motional inductance $L_{m}$ [25]. The capacitance $C_{0}$ results from the two electrodes that typically form a plate capacitor around the piezoelectric layer. Its dielectric losses are covered by the resistance $R_{0}$.

Apart from pulse mode operation, the two common continuous operation modes are often referred to as impedance-based measurement and oscillator-based measurement, respectively [25]. A closed-loop oscillator-based system contains a positive feedback network and generates an oscillating signal that is frequency-modulated (FM) by the measurand, while the signal’s amplitude is usually constant. On the contrary, open-loop impedance-based systems are characterized by amplitude-modulated (AM) and phase-modulated (PM) signals. Apart from practical differences between closed-loop and open-loop operation, FM and PM sensor systems are fully equivalent in terms of the minimum reachable limit of detection [26,27]. For open-loop operation, from previous studies it is known that the maximum sensitivity in AM mode is achieved by driving the resonator at a certain frequency [28] that is not equal to the frequency for which the sensitivity in PM mode is at its maximum [29]. However, a comprehensive analysis of the differences between AM and PM modes, especially with respect to the reachable frequency-dependent limits of detection, has not previously been performed and is presented in this article.

This article is organized as follows: Section 2 presents some fundamental characteristics of electromechanical resonators and introduces the properties of a magnetoelastic cantilever-type magnetic field sensor that is exemplarily utilized to illustrate the deduced relations. Section 3 presents the considered readout system utilized to reconstruct the time-varying physical quantity being measured. Both expressions for the frequency-dependent sensitivities, as well as frequency-dependent amplitude noise and phase noise, are deduced in Section 4 and Section 5, respectively. Based on these, Section 6 presents the resulting limits of detection. This article finishes with a summary of the findings in Section 7.

All expressions are given as a function of the two basic parameters of a resonator, the resonance frequency $f_{\mathrm{res}}$ and the quality factor *Q*. However, please note that several simplifying assumptions are made. It is assumed that the quality factor is independent of the measurand, and Q>100, which allows us to neglect a resonance detuning due to mechanical losses. All given results apply not only to cantilever-type sensors but to all types of micromechanical resonators that can be described by a damped harmonic oscillator.

## 2. Sensor

### 2.1. Electromechanical Resonator

An oscillating cantilever with the quality factor *Q*, the angular resonance frequency $\omega_{\mathrm{res}}=2\pi f_{\mathrm{res}}$, and the angular driving frequency ω=2πf can be described by the differential equation of motion of a damped driven harmonic oscillator

(1) $$\begin{array}{c} \frac{1}{\omega_{\mathrm{res}}^{2}}\frac{d^{2}z\left(t\right)}{dt^{2}}+\frac{1}{Q\omega_{\mathrm{res}}}\frac{dz(t)}{dt}+z\left(t\right)=\hat{Z}\cos\left(\omega t\right) \end{array}$$

where z(t) is the time-varying position of the cantilever tip and $\hat{Z}$ is the deflection amplitude [30] (p. 454). Solving for the differential equation’s steady state results in the well-known solution in the form of the complex-valued unitless frequency response of a damped driven harmonic oscillator [30] (p. 454) [31] (p. 427) [32]

(2) $$\begin{array}{c} G\left(f\right)=\frac{1}{1-{\left(\frac{f}{f_{\mathrm{res}}}\right)}^{2}+j\frac{f}{f_{\mathrm{res}}Q}}=\left|G\left(f\right)\right|\;\cdot \;e^{j\;\gamma (f)} \end{array}$$

where $j^{2}=-1$ is the imaginary number. For low-loss electromechanical resonators (Q>100) that are operated in the vicinity of their resonance frequency, the expression for the frequency response can be simplified, yielding

(3) $$\begin{array}{c} \tilde{G}\left(f\right)=\frac{1}{2\;\left(1-\frac{f}{f_{\mathrm{res}}}\right)+j\frac{1}{Q}}=\left|\tilde{G}\left(f\right)\right|\;\cdot \;e^{j\;\tilde{\gamma }\left(f\right)} \end{array}$$

with the amplitude frequency response

(4) $$\begin{array}{c} |\tilde{G}\left(f\right)|=\frac{1}{\sqrt{4\;{\left(1-\frac{f}{f_{\mathrm{res}}}\right)}^{2}+{\left(\frac{1}{Q}\right)}^{2}}} \end{array}$$

and the phase frequency response

(5) $$\begin{array}{c} \tilde{\gamma }\left(f\right)=\arctan\left(-\frac{1}{2Q\;\left(1-\frac{f}{f_{\mathrm{res}}}\right)}\right). \end{array}$$

**Figure 2:**
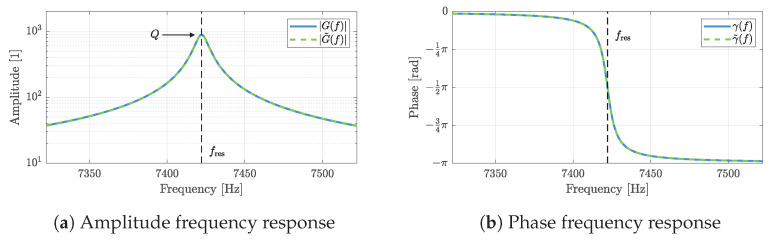
Comparison of amplitude (**a**) and phase frequency responses (**b**) between the original expression for the frequency response of a damped harmonic oscillator (Equation (2)) and the simplified expression according to Equation (3). In the vicinity of the resonance frequency, the simplified frequency response is a good approximation for quality factors Q>100. The depicted frequency responses are based on the parameters specified in Table 1.

Although the simplified frequency response (Equation (3)) is not a good approximation of the original frequency response (Equation (2)) far outside the resonance frequency, both the amplitude frequency response and the phase frequency response are well reproduced in the vicinity of the resonance frequency, as shown in Figure 2 for the exemplary cantilever that is described in more detail below in Section 2.3.

### 2.2. Sensor Admittance

The lumped-element equivalent electrical circuit of the electromechanical resonator has been introduced above and is depicted in Figure 1. Based on that network, the sensor’s admittance is simply given by

(6) $$\begin{array}{c} Y_{\mathrm{sensor}}\left(f\right)=Y_{m}\left(f\right)+j2\pi fC_{0}+\frac{1}{R_{0}\left(f\right)} \end{array}$$

where the resistance covering for the dielectric losses $R_{0}\left(f\right)={\left(2\pi fC_{0}\;\tan\delta_{0}\right)}^{-1}$ is frequency-dependent and defined by the dielectric loss factor $\tan\delta_{0}$ [33] (p. 568). However, in good approximation, $R_{0}$ can be considered constant if the sensor is operated in the vicinity of the resonance frequency. With both the resonance frequency $f_{\mathrm{res}}={\left(2\pi \sqrt{L_{m}C_{m}}\right)}^{-1}$ and the quality factor $Q=1/R_{m}\;\cdot \;\sqrt{L_{m}/C_{m}}$ as functions of the motional element’s values [33] (p. 402), the motional admittance

(7) $$\begin{array}{c} Y_{m}\left(f\right)={\left(R_{m}+j2\pi fL_{m}+\frac{1}{j2\pi fC_{m}}\right)}^{-1}=G\left(f\right)\;\cdot \;\frac{j}{QR_{m}}\;\cdot \;\frac{f}{f_{\mathrm{res}}} \end{array}$$

can be expressed as a function of the frequency response of a damped harmonic oscillator (Equation (2)). In the vicinity of the resonance frequency and for Q≫1, the motional admittance simplifies to

(8) $$\begin{array}{c} {\tilde{Y}}_{m}\left(f\right)=\tilde{G}\left(f\right)\;\cdot \;\frac{j}{QR_{m}}. \end{array}$$

Thus, a simplified expression for the sensor’s overall admittance yields

(9) $$\begin{array}{c} {\tilde{Y}}_{\mathrm{sensor}}\left(f\right)=\frac{1}{R_{0}\left(f_{\mathrm{res}}\right)}+j\left(2\pi fC_{0}+\frac{\tilde{G}\left(f\right)}{QR_{m}}\right). \end{array}$$

### 2.3. Exemplary Sensor

To illustrate the expressions derived in this article, the properties of an exemplary cantilever-type sensor are referred to. The micromechanical cantilever is designed for the detection of low-frequency magnetic fields (f<100 Hz) in the picotesla regime. It is based on a polysilicon cantilever of 3 mm length, 1 mm width, and 50 μm thickness. The bottom side is coated with 2 μm of magnetostrictive metal ((Fe_90_Co_10_)_78_Si_2_B_10_) that affects the cantilever’s resonance frequency by inducing an additional strain depending on an ambient magnetic field. On the top side, a film of 2 μm aluminum-nitride (AlN) piezoelectric material, surrounded by two electrodes, is deposited.

Further details about this sensor [10,11] with a focus on the MEMS fabrication process [34], the magnetic properties [35,36], and the readout and noise behavior [12,29,37,38] can be found in the literature. All electromechanical and magnetic properties required here are summarized in Table 1, where $B_{\mathrm{bias}}$ defines the constant magnetic operating point, and thus, the resonator’s initial state. This bias is used to maximize the magnetically induced resonance shift, i.e., the magnetic sensitivity $S_{\mathrm{mag}}$.

## 3. Sensor System

**Figure 3:**
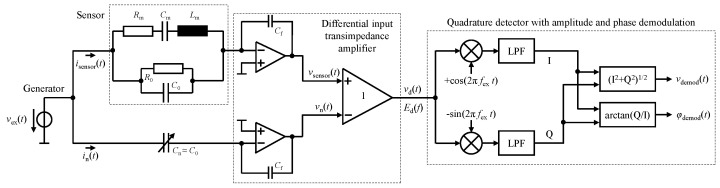
Electrical readout system for the measurement of a physical quantity (here, a magnetic flux density) that affects the resonance frequency of an electromechanical resonator. The sensor is driven in the vicinity of its resonance frequency by an electrical excitation signal $v_{\mathrm{ex}}\left(t\right)$, leading to an amplitude-modulated and phase-modulated signal $v_{d}\left(t\right)$ at the output of a differential input transimpedance amplifier. A subsequent quadrature detector performs both the amplitude demodulation and the phase demodulation.

For operating the cantilever-type sensor, an electrical system as depicted in Figure 3 is considered [29]. The basic principle is based on driving both the sensor and a capacitive branch to neutralize the parallel resonance due to the cantilever’s static capacitance $C_{0}$. For this, a differential input transimpedance/charge amplifier is utilized. Please note that the figure depicts a simplified illustration of such an amplifier. Refer to [39] for further details on differential input charge amplifiers. The excitation signal $v_{\mathrm{ex}}\left(t\right)={\hat{V}}_{\mathrm{ex}}\cos\left(2\pi f_{\mathrm{ex}}t\right)$ with the excitation amplitude ${\hat{V}}_{\mathrm{ex}}$ and the excitation frequency $f_{\mathrm{ex}}$ leads to currents through the sensor and through the neutralization branch that, in the frequency domain, can be described by $I_{\mathrm{sensor}}\left(f\right)=V_{\mathrm{ex}}\left(f\right)\;\cdot \;{\tilde{Y}}_{\mathrm{sensor}}\left(f\right)$ and $I_{n}\left(f\right)=V_{\mathrm{ex}}\left(f\right)\;\cdot \;j2\pi fC_{n}$ where $V_{\mathrm{ex}}\left(f\right)$ is the amplitude spectrum of $v_{\mathrm{ex}}\left(t\right)$.

By means of the transimpedance $T\left(f\right)=-{\left(j2\pi fC_{f}\right)}^{-1}$ both currents are transformed into the two voltage spectra

(10) $$\begin{array}{c} V_{\mathrm{sensor}}\left(f\right)=T\left(f\right)I_{\mathrm{sensor}}\left(f\right)=T\left(f\right)V_{\mathrm{ex}}\left(f\right)\;\cdot \;\left(\frac{1}{R_{0}\left(f_{\mathrm{res}}\right)}+j\left(2\pi fC_{0}+\frac{\tilde{G}\left(f\right)}{QR_{m}}\right)\right) \end{array}$$

and

(11) $$\begin{array}{c} V_{n}\left(f\right)=T\left(f\right)I_{n}\left(f\right)=T\left(f\right)V_{\mathrm{ex}}\left(f\right)\;\cdot \;j2\pi fC_{n} \end{array}$$

yielding a differential output voltage spectrum of

(12) $$\begin{array}{c} V_{d}\left(f\right)=V_{\mathrm{sensor}}\left(f\right)-V_{n}\left(f\right)=T\left(f\right)V_{\mathrm{ex}}\left(f\right)\left(\frac{1}{R_{0}\left(f_{\mathrm{res}}\right)}+j\frac{\tilde{G}\left(f\right)}{QR_{m}}\right) \end{array}$$

if the neutralization capacitance $C_{n}$ is matched to the sensor’s static capacitance $C_{0}$. The dielectric losses taken into account by $R_{0}$ are important for the noise analysis in Section 5 below. However, due to its high resistance (compare Table 1), the impact of $R_{0}$ can be neglected here, yielding

(13) $$\begin{array}{c} V_{d}\left(f\right)=j\;T\left(f\right)V_{\mathrm{ex}}\left(f\right)\;\frac{\tilde{G}\left(f\right)}{QR_{m}}=-\frac{V_{\mathrm{ex}}\left(f\right)\cdot \tilde{G}\left(f\right)}{2\pi f_{\mathrm{ex}}C_{f}\cdot QR_{m}}. \end{array}$$

This spectrum corresponds with the time domain signal

(14) $$\begin{array}{c} v_{d}\left(t\right)=-\frac{{\hat{V}}_{\mathrm{ex}}}{2\pi f_{\mathrm{ex}}C_{f}\cdot QR_{m}}\;\cdot \;\left|\tilde{G}\left(f_{\mathrm{ex}},B\left(t\right)\right)\right|\;\cdot \;\cos\left(2\pi f_{\mathrm{ex}}t+\tilde{\gamma }\left(f_{\mathrm{ex}},B\left(t\right)\right)\right) \end{array}$$

when considering that the resonator, i.e., $\tilde{G}\left(f\right)$ (Equation (3)), is affected by an ambient magnetic flux density $B\left(t\right)=B_{\mathrm{bias}}+B_{x}\left(t\right)$ that consists of the constant bias to set the sensor’s magnetic operating point (initial state) and a time-dependent magnetic signal $B_{x}\left(t\right)$ to be measured. Both the magnitude

(15) $$\begin{array}{c} |\tilde{G}\left(f_{\mathrm{ex}},B\left(t\right)\right)\left|=\right|\tilde{G}\left(f_{\mathrm{ex}},B_{\mathrm{bias}}\right)|\;\cdot \;\left[1+\underset{S_{\mathrm{AM}}}{\underset{︸}{\frac{\partial |\tilde{G}\left(f\right)|}{|\tilde{G}\left(f_{\mathrm{ex}},B_{\mathrm{bias}}\right)|\;\partial B_{x}}{|}_{f=f_{\mathrm{ex}}}}}\;\cdot \;B_{x}\left(t\right)\right] \end{array}$$

and the phase

(16) $$\begin{array}{c} \tilde{\gamma }\left(f_{\mathrm{ex}},B\left(t\right)\right)=\tilde{\gamma }\left(f_{\mathrm{ex}},B_{\mathrm{bias}}\right)+\underset{S_{\mathrm{PM}}}{\underset{︸}{\frac{\partial \tilde{\gamma }\left(f\right)}{\partial B_{x}}{|}_{f=f_{\mathrm{ex}}}}}\;\cdot \;B_{x}\left(t\right). \end{array}$$

of the resonator’s frequency response $\tilde{G}\left(f\right)$ can be described by a sum of a static term, defined by $B_{\mathrm{bias}}$, and a (fractional) dynamic term that changes with $B_{x}\left(t\right)$. The coefficients of the dynamic terms are referred to as sensitivities in amplitude mode ($S_{\mathrm{AM}}$) and phase mode ($S_{\mathrm{PM}}$), respectively. Expressions are given, and more details are discussed, in Section 4. Considering additional random fractional amplitude fluctuations α(t) and random phase fluctuations φ(t) [40] (p. 3), the total differential voltage is given by

(17) $$\begin{array}{cc} v_{d}\left(t\right) & =\overset{{\hat{V}}_{d}}{\overset{︷}{-\frac{{\hat{V}}_{\mathrm{ex}}\;\cdot \;\left|\tilde{G}\left(f_{\mathrm{ex}},B_{\mathrm{bias}}\right)\right|}{2\pi f_{\mathrm{ex}}C_{f}\;\cdot \;QR_{m}}}}\;\cdot \;\left[1+\overset{\mathrm{amplitude}\;\mathrm{modulation}}{\overset{︷}{S_{\mathrm{AM}}B_{x}\left(t\right)}}+\overset{\mathrm{amplitude}\;\mathrm{noise}}{\overset{︷}{\alpha (t)}}\right] \end{array}$$

(18) $$\begin{array}{cc} \; & \;\;\cdot \;\cos\left(2\pi f_{\mathrm{ex}}t+\tilde{\gamma }\left(f_{\mathrm{ex}},B_{\mathrm{bias}}\right)+\underset{\mathrm{phase}\;\mathrm{modulation}}{\underset{︸}{S_{\mathrm{PM}}B_{x}\left(t\right)}}+\underset{\mathrm{phase}\;\mathrm{noise}}{\underset{︸}{\varphi (t)}}\right). \end{array}$$

As depicted in Figure 3, the modulated signal $v_{d}\left(t\right)$ is demodulated employing a quadrature detector, yielding the amplitude-demodulated voltage

(19) $$\begin{array}{c} v_{\mathrm{demod}}\left(t\right)=\frac{{\hat{V}}_{d}}{2}\;\cdot \;\left[1+S_{\mathrm{AM}}B_{x}\left(t\right)+\alpha \left(t\right)\right] \end{array}$$

and the phase-demodulated signal

(20) $$\begin{array}{c} \varphi_{\mathrm{demod}}\left(t\right)=\tilde{\gamma }\left(f_{\mathrm{ex}},B_{\mathrm{bias}}\right)+S_{\mathrm{PM}}B_{x}\left(t\right)+\varphi \left(t\right) \end{array}$$

if assuming that the two low-pass filters (LPF) ideally suppress the frequency components around $2f_{\mathrm{ex}}$.

## 4. Sensitivity

The sensitivity of resonant micromechanical sensors is composed of several partial sensitivities, which are described and derived below.

### 4.1. Functional Sensitivity

The functional sensitivity covers the relationship between the physical measurand to be detected and the frequency detuning of the micromechanical resonator. In the case of the magnetic field sensor used in this article to illustrate the results, the functional sensitivity is referred to as the magnetic sensitivity

(21) $$\begin{array}{c} S_{\mathrm{mag}}\left(B_{\mathrm{bias}}\right)=\frac{\partial f_{\mathrm{res}}}{\partial B_{x}} \end{array}$$

with the physical dimension Hz/T. For other types of resonant sensors, the functional sensitivity could have, for example, the physical dimensions of Hz/K (temperature sensor), Hz/N (force sensor), or Hz/kg (mass sensor). Note that the functional sensitivity only depends on the sensor’s initial state, here set by the constant magnetic bias $B_{\mathrm{bias}}$.

### 4.2. Electrical Sensitivity

**Figure 4:**
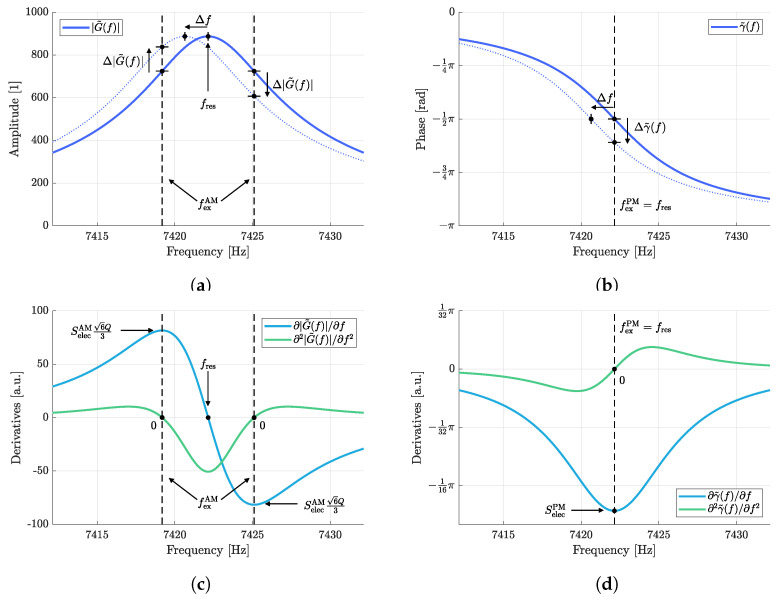
Illustration of the relations between shifted amplitude (**a**) and phase (**b**) frequency responses due to a detuned resonance frequency and the corresponding electrical sensitivities. Highest sensitivities are given by the corresponding first derivative at those frequencies at which the second derivatives are zero (**c**,**d**). These frequencies are referred to as the optimum excitation frequencies. The plots are based on the sensor parameters specified in Table 1.

In addition to the influence of the physical measurand on the sensor’s resonance frequency, an additional sensitivity is required to characterize how the frequency detuning changes the sensor signal. Two sensitivities are required for this purpose, one covering the influence on the amplitude and the other the influence on the phase, where the two sensitivities are referred to as electrical sensitivities. The Figure 4a and Figure 4b illustrate the change in amplitude $\Delta |\tilde{G}\left(f\right)|$ and in phase $\Delta \tilde{\gamma }\left(f\right)$ due to a detuned resonance frequency by Δf. To determine the optimal excitation frequencies and the resulting sensitivities, the two first derivatives

(22) $$\begin{array}{c} \frac{\partial |\tilde{G}\left(f\right)|}{\partial f}=-\frac{4\;(f-f_{\mathrm{res}})}{f_{\mathrm{res}}^{2}\;{\left(4\;{\left(\frac{f}{f_{\mathrm{res}}}-1\right)}^{2}+\frac{1}{Q^{2}}\right)}^{\frac{3}{2}}} \end{array}$$

and

(23) $$\begin{array}{c} \frac{\partial \tilde{\gamma }\left(f\right)}{\partial f}=-\frac{2\;Qf_{\mathrm{res}}}{4Q^{2}\;{\left(f-f_{\mathrm{res}}\right)}^{2}+f_{\mathrm{res}}^{2}} \end{array}$$

as well as the two second derivatives

(24) $$\begin{array}{c} \frac{\partial^{2}\left|\tilde{G}\left(f\right)\right|}{\partial f^{2}}=\frac{32\;Q^{2}f^{2}-64\;Q^{2}ff_{\mathrm{res}}+32\;Q^{2}f_{\mathrm{res}}^{2}-4\;f_{\mathrm{res}}^{2}}{Q^{2}f_{\mathrm{res}}^{4}\;{\left(4\;{\left(\frac{f}{f_{\mathrm{res}}}-1\right)}^{2}+\frac{1}{Q^{2}}\right)}^{\frac{5}{2}}} \end{array}$$

and

(25) $$\begin{array}{c} \frac{\partial^{2}\tilde{\gamma }\left(f\right)}{\partial f^{2}}=\frac{16\;Q^{3}f_{\mathrm{res}}\;\left(f-f_{\mathrm{res}}\right)}{{\left(4\;Q^{2}f^{2}-8\;Q^{2}ff_{\mathrm{res}}+4\;Q^{2}f_{\mathrm{res}}^{2}+f_{\mathrm{res}}^{2}\right)}^{2}} \end{array}$$

of the amplitude frequency response $|\tilde{G}\left(f\right)|$ (Equation (4)) and the phase frequency response $\tilde{\gamma }\left(f\right)$ (Equation (5)), respectively, are required. These derivatives are depicted in Figure 4c,d and visualize that the maximum influence on the signal, i.e., the highest sensitivity, is given by the corresponding first derivative at those frequencies at which the second derivatives are zero. These frequencies are referred to as the optimum excitation frequencies.

Determining the roots of the second derivatives by solving $\partial^{2}\left|\tilde{G}\left(f\right)\right|/\partial f^{2}=0$ for *f* and $\partial^{2}\tilde{\gamma }\left(f\right)/\partial f^{2}=0$ for *f*, respectively, yields the two optimum excitation frequencies for the amplitude mode

(26) $$\begin{array}{c} f_{\mathrm{ex}}^{\mathrm{AM}}=f_{\mathrm{res}}\left(1\mp \frac{1}{\sqrt{8}\;Q}\right) \end{array}$$

that agree with reported results in [28] and the optimum excitation frequency for the phase mode

(27) $$\begin{array}{c} f_{\mathrm{ex}}^{\mathrm{PM}}=f_{\mathrm{res}}. \end{array}$$

At these optimum excitation frequencies, the values of the electrical sensitivities are given by

(28) $$\begin{array}{c} S_{\mathrm{elec}}^{\mathrm{AM}}=\frac{\partial |\tilde{G}\left(f\right)|}{\overset{|\tilde{G}\left(f_{\mathrm{ex}}^{\mathrm{AM}}\right)|}{\underset{\frac{\sqrt{6}\;Q}{3}}{︸}}\;\partial f}{|}_{f=f_{\mathrm{ex}}^{\mathrm{AM}}}=\pm \frac{4\sqrt{3}}{9}\frac{Q^{2}}{f_{\mathrm{res}}}\;\cdot \;\frac{3}{\sqrt{6}\;Q}=\pm \frac{2\sqrt{2}}{3}\frac{Q}{f_{\mathrm{res}}} \end{array}$$

and

(29) $$\begin{array}{c} S_{\mathrm{elec}}^{\mathrm{PM}}=\frac{\partial \tilde{\gamma }\left(f\right)}{\partial f}{|}_{f=f_{\mathrm{ex}}^{\mathrm{PM}}}=-\frac{2Q}{f_{\mathrm{res}}}. \end{array}$$

### 4.3. Dynamic Sensitivity

The functional sensitivity combined with the electrical sensitivity already gives the sensor’s total sensitivity with regard to static measurands, i.e., for a constant magnetic flux density. However, for dynamic measurements, i.e., if the signal to be measured is of the general form $B_{x}\left(t\right)={\hat{B}}_{x}\cos\left(2\pi f_{x}t\right)$, the sensor’s sensitivity decreases for higher frequencies $f_{x}$ as shown in measurements [12]. This effect is taken into account by the so-called dynamic sensitivity. In normalized form, the dynamic sensitivity, which typically exhibits a low-pass behavior, can be calculated according to

(30) $$\begin{array}{c} S_{\mathrm{dyn}}\left(f_{x}\right)=\frac{1}{2}\;\cdot \;\left(\frac{\tilde{G}\left(f_{\mathrm{ex}}+f_{x}\right)}{\tilde{G}\left(f_{\mathrm{ex}}\right)}+\frac{{\tilde{G}}^{*}\left(f_{\mathrm{ex}}-f_{x}\right)}{{\tilde{G}}^{*}\left(f_{\mathrm{ex}}\right)}\right) \end{array}$$

where ${}^{*}$ denotes a complex conjugate [41]. Because the optimum excitation frequencies differ in amplitude mode (Equation (26)) and in phase mode (Equation (26)), the AM dynamic sensitivity

(31) $$\begin{array}{cc} S_{\mathrm{dyn}}^{\mathrm{AM}}\left(f_{x}\right) & =S_{\mathrm{dyn}}\left(f_{x}\right){|}_{f_{\mathrm{ex}}=f_{\mathrm{ex}}^{\mathrm{AM}}}=\left|S_{\mathrm{dyn}}^{\mathrm{AM}}\left(f_{x}\right)\right|\;\cdot \;e^{j\;\varphi_{\mathrm{dyn}}^{\mathrm{AM}}\left(f_{x}\right)}\\ \; & =\frac{f_{\mathrm{res}}^{2}\sqrt{81\;f_{\mathrm{res}}^{4}+144\;Q^{2}f_{\mathrm{res}}^{2}f_{x}^{2}+576\;Q^{4}f_{x}^{4}}}{64\;Q^{4}f_{x}^{4}+16\;Q^{2}f_{\mathrm{res}}^{2}f_{x}^{2}+9\;f_{\mathrm{res}}^{4}}\;\cdot \;e^{j\;\left(\arctan\left(\frac{1}{\sqrt{8}}+\frac{9}{\sqrt{512}}\;{\left(\frac{f_{\mathrm{res}}}{Qf_{x}}\right)}^{2}\right)-\frac{\pi }{2}\right)} \end{array}$$

also differs from the PM dynamic sensitivity

(32) $$\begin{array}{cc} S_{\mathrm{dyn}}^{\mathrm{PM}}\left(f_{x}\right) & =S_{\mathrm{dyn}}\left(f_{x}\right){|}_{f_{\mathrm{ex}}=f_{\mathrm{ex}}^{\mathrm{PM}}}=\left|S_{\mathrm{dyn}}^{\mathrm{PM}}\left(f_{x}\right)\right|\;\cdot \;e^{j\;\varphi_{\mathrm{dyn}}^{\mathrm{PM}}\left(f_{x}\right)}\\ \; & =\frac{1}{\sqrt{1+{\left(\frac{2Qf_{x}}{f_{\mathrm{res}}}\right)}^{2}}}\;\cdot \;e^{j\;\arctan\left(-\frac{2Qf_{x}}{f_{\mathrm{res}}}\right)}. \end{array}$$

**Figure 5:**
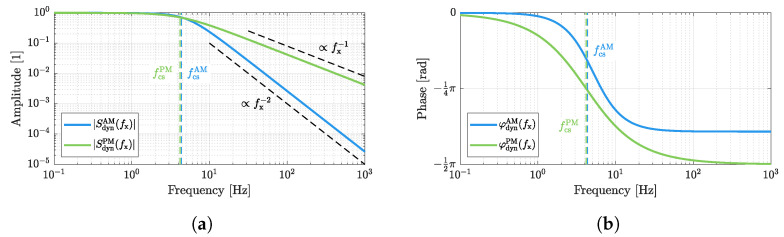
Illustrations of the dynamic sensitivities for amplitude mode and phase mode. The −3 dB cutoff frequencies are almost identical, whereas the slopes of the amplitude frequency responses differ. The plots are based on the sensor parameters specified in Table 1. (**a**) amplitude frequency responses, (**b**) phase frequency responses.

For both operation modes, the amplitude frequency responses and the phase frequency responses are depicted in Figure 5. The −3 dB cutoff frequencies of

(33) $$\begin{array}{c} f_{\mathrm{cs}}^{\mathrm{AM}}=\frac{f_{\mathrm{res}}\;\sqrt{2}\sqrt{\sqrt{10}-1}}{4Q}\approx \frac{f_{\mathrm{res}}}{2Q} \end{array}$$

and

(34) $$\begin{array}{c} f_{\mathrm{cs}}^{\mathrm{PM}}=\frac{f_{\mathrm{res}}}{2Q} \end{array}$$

are almost identical. However, the amplitude response above the cutoff frequency decreases proportional to $f_{x}^{-2}$ (−40 dB/decade) for AM, whereas the decrease for PM is only proportional to $f_{x}^{-1}$ (−20 dB/decade).

### 4.4. Overall Sensitivity

For a certain operating point of the sensor, defined by the constant bias $B_{\mathrm{bias}}$, the overall sensitivities are given by the products of the individual sensitivities and yield

(35) $$\begin{array}{cc} S_{\mathrm{AM}}\left(f_{x}\right) & =S_{\mathrm{mag}}\;\cdot \;S_{\mathrm{elec}}^{\mathrm{AM}}\;\cdot \;S_{\mathrm{dyn}}^{\mathrm{AM}}\left(f_{x}\right) \end{array}$$

(36) $$\begin{array}{cc} \; & =\pm S_{\mathrm{mag}}\;\cdot \;\frac{2\sqrt{2}}{3}\frac{Q}{f_{\mathrm{res}}}\;\cdot \;\frac{f_{\mathrm{res}}^{2}\;\left(9\;f_{\mathrm{res}}^{2}+8\;Q^{2}f_{x}^{2}-j\;\sqrt{512}\;Q^{2}f_{x}^{2}\right)}{64\;Q^{4}f_{x}^{4}+16\;Q^{2}f_{\mathrm{res}}^{2}f_{x}^{2}+9\;f_{\mathrm{res}}^{4}} \end{array}$$

and

(37) $$\begin{array}{cc} S_{\mathrm{PM}}\left(f_{x}\right) & =S_{\mathrm{mag}}\;\cdot \;S_{\mathrm{elec}}^{\mathrm{PM}}\;\cdot \;S_{\mathrm{dyn}}^{\mathrm{PM}}\left(f_{x}\right) \end{array}$$

(38) $$\begin{array}{cc} \; & =-S_{\mathrm{mag}}\;\cdot \;\frac{2Q}{f_{\mathrm{res}}}\;\cdot \;\frac{1-j\frac{2Qf_{x}}{f_{\mathrm{res}}}}{1+{\left(\frac{2Qf_{x}}{f_{\mathrm{res}}}\right)}^{2}} \end{array}$$

for amplitude mode operation and phase mode operation, respectively.

## 5. Noise

A micromechanical sensor comprises several loss mechanisms which, according to the fluctuation–dissipation theorem [42], correspond with noise. In general, such losses can be taken into account in an electrical equivalent circuit in the form of dissipative elements, i.e., by electrical resistors. For piezoelectric resonators, and in accordance with the two resistors in the sensor’s electrical equivalent circuit in Figure 1, the loss mechanisms can be divided into two types.

Dielectric losses are affiliated with the sensor’s capacitor-like structure, i.e., to the piezoelectric material acting as the dielectric medium. These dielectric losses are considered by the loss factor $\tan\delta_{0}$ with reported values for thin-film piezoelectric materials as low as, e.g., $2.5\;\cdot \;10^{-4}$ (aluminum-nitride, AlN) [43], $1.3\;\cdot \;10^{-3}$ (aluminum-scandium-nitride, AlScN) [44], and $4\;\cdot \;10^{-3}$ (lead-zirconate-titanate, PZT) [45]. The exemplary sensor referred to in this article exhibits a value of $\tan\delta_{0}=5\;\cdot \;10^{-3}$, thus resulting in a resistance in parallel to the static capacitance $C_{0}$ with a value in the vicinity of the resonance frequency of $R_{0}\left(f_{\mathrm{res}}\right)={\left(2\pi f_{\mathrm{res}}C_{0}\;\tan\delta_{0}\right)}^{-1}\approx 97.5\;M\Omega$.

The predominant loss mechanism of micromechanical cantilevers under atmospheric pressure is usually air damping, commonly referred to as viscous damping. In addition, e.g., thermoelastic friction intrinsic to the solid structure, support losses, surface losses, and mounting losses may further attenuate the cantilever’s deflection, also expressed by its quality factor *Q* [46]. In the electrical equivalent circuit model, these losses are taken into account by the motional resistance $R_{m}$.

In addition to these standard loss mechanisms, depending on the type of sensor, the special functional layer may introduce additional dominant losses, e.g., in the case of magnetostrictive films [29,38]. Such losses and the associated noise are not considered here. However, they can be taken into account using the same approach as shown below if necessary. Furthermore, the noise of the electronics is also neglected at this point, because corresponding amplifiers can generally be realized with very low noise [47], and likewise, at least the phase noise of the generator can be sufficiently suppressed [48].

### 5.1. Voltage Noise

**Figure 6:**
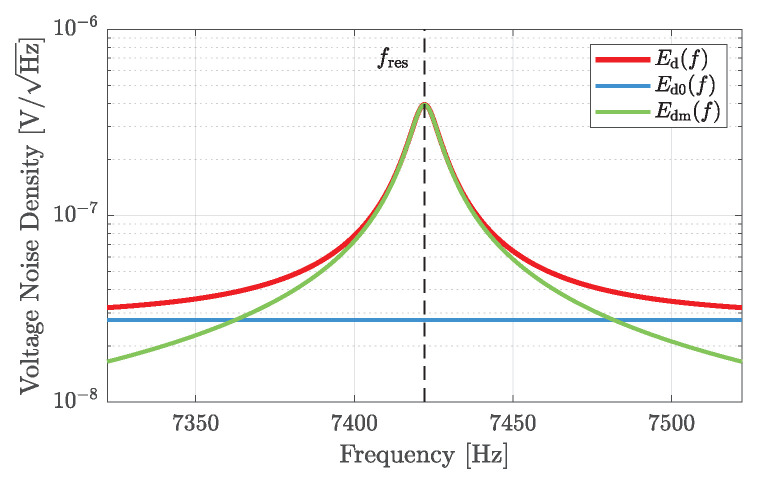
Noise contributions of the sensor to the overall voltage noise density at the output of the differential input transimpedance amplifier for the exemplary sensor described in Table 1, for feedback capacitances of $C_{f}=10\;\mathrm{pF}$, and without any excitation signal. Near the resonance frequency, the total noise is dominated by the thermal mechanical noise $E_{\mathrm{dm}}$, whereas the thermal electrical noise $E_{d0}$ becomes relevant only at some distance from $f_{\mathrm{res}}$.

Both the thermal electrical Johnson–Nyquist noise of the piezoelectric material acting as the capacitor’s dielectric $E_{0}\left(f\right)=\sqrt{4k_{B}T_{0}R_{0}}$ and the thermal mechanical noise of the micromechanical resonator $E_{m}\left(f\right)=\sqrt{4k_{B}T_{0}R_{m}}$ can be accurately predicted [38,47,49] based on the sensor’s electrical impedance. For the sensor readout system as depicted in Figure 3, the thermal voltage noise contributions related to the output of the differential input transimpedance amplifier are given by

(39) $$\begin{array}{c} E_{d0}\left(f\right)=\frac{\left|T(f_{\mathrm{res}})\right|}{R_{0}\left(f_{\mathrm{res}}\right)}\sqrt{4k_{B}T_{0}R_{0}\left(f_{\mathrm{res}}\right)}=\frac{1}{\pi f_{\mathrm{res}}C_{f}}\;\sqrt{\frac{k_{B}T_{0}}{R_{0}\left(f_{\mathrm{res}}\right)}}=\mathrm{const}. \end{array}$$

and

(40) $$\begin{array}{c} E_{\mathrm{dm}}\left(f\right)=\left|T\left(f_{\mathrm{res}}\right){\tilde{Y}}_{m}\left(f\right)\right|\sqrt{4k_{B}T_{0}R_{m}}=\frac{|\tilde{G}\left(f\right)|}{\pi f_{\mathrm{res}}C_{f}\;Q}\;\sqrt{\frac{k_{B}T_{0}}{R_{m}}}\neq \mathrm{const}. \end{array}$$

if, as above, T(f) and $R_{0}\left(f\right)$ are assumed as constant in the vicinity of the resonance frequency, where $k_{B}\approx 1.381\;\cdot \;10^{-23}\;J/K$ is the Boltzmann constant and $T_{0}=290K$ the room temperature [29]. As expected, the thermal mechanical noise $E_{\mathrm{dm}}$ of the resonator is weighted by the magnitude of the frequency response of the damped harmonic oscillator, whereas the thermal electrical noise of the piezoelectric material is independent of the frequency [49]. Both statistically independent voltage noise densities of the sensor add up to

(41) $$\begin{array}{c} E_{d}\left(f\right)=\sqrt{E_{d0}^{2}\left(f\right)+E_{\mathrm{dm}}^{2}\left(f\right)} \end{array}$$

and are depicted in Figure 6 for the exemplary sensor without any excitation signal (${\hat{V}}_{\mathrm{ex}}=0$).

### 5.2. Amplitude Noise and Phase Noise

To determine the relationship between the introduced voltage noise densities and the power spectral densities (PSD) $S_{\alpha }\left(f\right)$ and $S_{\varphi }\left(f\right)$ of the random amplitude fluctuations α(t) and random phase fluctuations φ(t), respectively, Equation (18) is written as

(42) $$\begin{array}{c} v_{d}\left(t\right)={\hat{V}}_{d}\;\cdot \;[1+\underset{\alpha (t)}{\underset{︸}{\hat{\alpha }\left(f_{x}\right)\;\cos\left(2\pi f_{x}t\right)}}]\;\cdot \;\cos(2\pi f_{\mathrm{ex}}t+\underset{\varphi (t)}{\underset{︸}{\hat{\varphi }\left(f_{x}\right)\;\cos\left(2\pi f_{x}t\right)}}) \end{array}$$

in which the measurement signal $B_{x}\left(t\right)$ and the static phase offset, which are both irrelevant to the noise, are neglected. In this equation, one amplitude noise component $\hat{\alpha }$ and one phase noise component $\hat{\varphi }$, both at the frequency $f_{x}$, represent other spectral components that can be taken into account by linear superposition. Based on basic trigonometric identities, Equation (42) can be rearranged into

(43) $$\begin{array}{cc} v_{d}\left(t\right) & ={\hat{V}}_{d}\;\cdot \;\cos\left(2\pi f_{\mathrm{ex}}t\right) \end{array}$$

(44) $$\begin{array}{cc} \; & \;+\frac{{\hat{V}}_{d}\;\hat{\alpha }\left(f_{x}\right)}{2}\;\cdot \;\left[\cos\left(2\pi (f_{\mathrm{ex}}-f_{x})t\right)+\cos\left(2\pi (f_{\mathrm{ex}}+f_{x})t\right)\right] \end{array}$$

(45) $$\begin{array}{cc} \; & \;-\frac{{\hat{V}}_{d}\;\hat{\varphi }\left(f_{x}\right)}{2}\;\cdot \;\left[\sin\left(2\pi (f_{\mathrm{ex}}-f_{x})t\right)+\sin\left(2\pi (f_{\mathrm{ex}}+f_{x})t\right)\right] \end{array}$$

revealing the typical structures of an amplitude-modulated signal and a narrow-band small-signal phase-modulated signal, respectively, with a carrier at $f_{\mathrm{ex}}$ and symmetrical sidebands at $f_{\mathrm{ex}}\pm f_{x}$. Following the concept of noise sidebands [50] (p. 243), both carrier-to-noise sideband ratios

(46) $$\begin{array}{c} \frac{{\hat{V}}_{d}}{\frac{{\hat{V}}_{d}\;\hat{\alpha }\left(f_{x}\right)}{2}}=\frac{{\hat{V}}_{d}}{\frac{{\hat{V}}_{d}\;\hat{\varphi }\left(f_{x}\right)}{2}}=\frac{2}{\hat{\alpha }\left(f_{x}\right)}=\frac{2}{\hat{\varphi }\left(f_{x}\right)}=\frac{\frac{{\hat{V}}_{d}}{\sqrt{2}}}{\frac{1}{2}\sqrt{E_{d}^{2}\left(f_{\mathrm{ex}}-f_{x}\right)+E_{d}^{2}\left(f_{\mathrm{ex}}+f_{x}\right)}\;\sqrt{\Delta f}} \end{array}$$

are equal to the carrier-to-voltage noise ratio where the additional factor $\sqrt{\Delta f}$ transforms the voltage noise density $E_{d}$ into an effective voltage noise in the bandwidth Δf. Equation (46) directly yields the PSD of random amplitude fluctuations

(47) $$\begin{array}{c} S_{\alpha }\left(f_{x}\right)={\left(\frac{\hat{\alpha }\left(f_{x}\right)}{\sqrt{2}\sqrt{\Delta f}}\right)}^{2}=\frac{E_{d}^{2}\left(f_{\mathrm{ex}}^{\mathrm{AM}}-f_{x}\right)+E_{d}^{2}\left(f_{\mathrm{ex}}^{\mathrm{AM}}+f_{x}\right)}{{\hat{V}}_{d}^{2}}=S_{\alpha 0}\left(f_{x}\right)+S_{\alpha m}\left(f_{x}\right) \end{array}$$

in units of 1/Hz and the PSD of random phase fluctuations

(48) $$\begin{array}{c} S_{\varphi }\left(f_{x}\right)={\left(\frac{\hat{\varphi }\left(f_{x}\right)}{\sqrt{2}\sqrt{\Delta f}}\right)}^{2}=\frac{E_{d}^{2}\left(f_{\mathrm{ex}}^{\mathrm{PM}}-f_{x}\right)+E_{d}^{2}\left(f_{\mathrm{ex}}^{\mathrm{PM}}+f_{x}\right)}{{\hat{V}}_{d}^{2}}=S_{\varphi 0}\left(f_{x}\right)+S_{\varphi m}\left(f_{x}\right) \end{array}$$

in units of ${\mathrm{rad}}^{2}/\mathrm{Hz}$. Each PSD is composed of a constant component due to the thermal electrical noise $E_{d0}$ of the piezoelectric material

(49) $$\begin{array}{c} S_{\alpha 0}\left(f_{x}\right)=\frac{12\;k_{B}T_{0}R_{m}^{2}{\left(\frac{\sqrt{2}}{4Q}-1\right)}^{2}}{{\hat{V}}_{\mathrm{ex}}^{2}\;R_{0}} \end{array}$$

(50) $$\begin{array}{c} S_{\varphi 0}\left(f_{x}\right)=\frac{8k_{B}T_{0}R_{m}^{2}}{{\hat{V}}_{\mathrm{ex}}^{2}\;R_{0}} \end{array}$$

and a frequency-dependent component

(51) $$\begin{array}{c} S_{\alpha m}\left(f_{x}\right)=\frac{3\;k_{B}T_{0}R_{m}f_{\mathrm{res}}^{2}\left(8\;Q^{2}f_{x}^{2}+3\;f_{\mathrm{res}}^{2}\right)\left(8\;Q^{2}-4\sqrt{2}\;Q+1\right)}{{\hat{V}}_{\mathrm{ex}}^{2}Q^{2}\;\left(64\;Q^{4}f_{x}^{4}+16\;Q^{2}f_{\mathrm{res}}^{2}f_{x}^{2}+9\;f_{\mathrm{res}}^{4}\right)} \end{array}$$

(52) $$\begin{array}{c} S_{\varphi m}\left(f_{x}\right)=\frac{8k_{B}T_{0}R_{m}}{{\hat{V}}_{\mathrm{ex}}^{2}\left({\left(\frac{2Qf_{x}}{f_{\mathrm{res}}}\right)}^{2}+1\right)} \end{array}$$

due to the thermal mechanical noise $E_{\mathrm{dm}}$ of the resonator. As illustrated in Figure 7, $S_{\alpha m}$ and $S_{\varphi m}$ decrease with 20 dB/decade for frequencies $f_{x}$ above the respective −3 dB cutoff frequency of $f_{\mathrm{cn}}^{\mathrm{AM}}=f_{\mathrm{res}}\;\sqrt{2}\sqrt{\sqrt{13}+2}/\left(4Q\right)$ and $f_{\mathrm{cn}}^{\mathrm{PM}}=f_{\mathrm{res}}/\left(2Q\right)$. In contrast to parametric noise, all four noise components involved here are additive noise. Such noise is characterized by the fact that it can be reduced by increasing the excitation amplitude ${\hat{V}}_{\mathrm{ex}}$.

## 6. Detectivity

The frequency-dependent noise floor of a sensor system is usually given by a spectral density that is related to the unit of the physical quantity to be detected. For a physical quantity with the arbitrary unit au, the representation of the sensor system’s noise floor could be given as a PSD of the fluctuations of the arbitrary quantity in units of ${\mathrm{au}}^{2}/\mathrm{Hz}$. However, in general, it is more common to use the amplitude spectral density (ASD) of the fluctuations of the arbitrary quantity in units of $\mathrm{au}/\sqrt{\mathrm{Hz}}$, referred to as the limit of detection (LOD) or detectivity [26]. For the magnetic field sensor system considered in this article, the LOD is given in units of $T/\sqrt{\mathrm{Hz}}$; thus, it is also referred to as equivalent magnetic noise floor.

Depending on the operation mode, the LOD is given by the ratio of the ASD of random amplitude fluctuations and the overall amplitude sensitivity

(53) $$\begin{array}{c} {\mathrm{LOD}}_{\mathrm{AM}}\left(f_{x}\right)=\frac{\sqrt{S_{\alpha }\left(f_{x}\right)}}{|S_{\mathrm{AM}}\left(f_{x}\right)|}=\frac{\sqrt{S_{\alpha 0}\left(f_{x}\right)+S_{\alpha m}\left(f_{x}\right)}}{|S_{\mathrm{AM}}\left(f_{x}\right)|} \end{array}$$

or by the ratio of the ASD of random phase fluctuations and the overall phase sensitivity

(54) $$\begin{array}{c} {\mathrm{LOD}}_{\mathrm{PM}}\left(f_{x}\right)=\frac{\sqrt{S_{\varphi }\left(f_{x}\right)}}{|S_{\mathrm{PM}}\left(f_{x}\right)|}=\frac{\sqrt{S_{\varphi 0}\left(f_{x}\right)+S_{\varphi m}\left(f_{x}\right)}}{|S_{\mathrm{PM}}\left(f_{x}\right)|}. \end{array}$$

In each case, the detectivity is limited by the same two physical noise sources. The limit due to the thermal electrical noise of the dielectric material yields

(55) $$\begin{array}{c} {\mathrm{LOD}}_{\mathrm{AM}0}\left(f_{x}\right)=\frac{\sqrt{S_{\alpha 0}\left(f_{x}\right)}}{|S_{\mathrm{AM}}\left(f_{x}\right)|}=\frac{R_{m}\;\sqrt{6k_{B}T_{0}}\;\left(4Q-\sqrt{2}\right)\sqrt{64\;Q^{4}f_{x}^{4}+16\;Q^{2}f_{x}^{2}f_{\mathrm{res}}^{2}+9f_{\mathrm{res}}^{4}}}{8\;S_{\mathrm{mag}}{\hat{V}}_{\mathrm{ex}}Q^{2}f_{\mathrm{res}}\sqrt{R_{0}}} \end{array}$$

and

(56) $$\begin{array}{c} {\mathrm{LOD}}_{\mathrm{PM}0}\left(f_{x}\right)=\frac{\sqrt{S_{\varphi 0}\left(f_{x}\right)}}{|S_{\mathrm{PM}}\left(f_{x}\right)|}=\frac{R_{m}\;\sqrt{2k_{B}T_{0}}\;\sqrt{4Q^{2}f_{x}^{2}+f_{\mathrm{res}}^{2}}}{S_{\mathrm{mag}}{\hat{V}}_{\mathrm{ex}}Q\;\sqrt{R_{0}}} \end{array}$$

whereas the limit due to the thermal mechanical noise of the resonator is given by

(57) $$\begin{array}{c} {\mathrm{LOD}}_{\mathrm{AMm}}\left(f_{x}\right)=\frac{\sqrt{S_{\alpha m}\left(f_{x}\right)}}{|S_{\mathrm{AM}}\left(f_{x}\right)|}=\frac{\sqrt{6k_{B}T_{0}R_{m}}\;\sqrt{8\;Q^{2}f_{x}^{2}+3\;f_{\mathrm{res}}^{2}}\;\sqrt{8\;Q^{2}-4\sqrt{2}\;Q+1}}{4\;S_{\mathrm{mag}}{\hat{V}}_{\mathrm{ex}}Q^{2}} \end{array}$$

and

(58) $$\begin{array}{c} {\mathrm{LOD}}_{\mathrm{PMm}}\left(f_{x}\right)={\mathrm{LOD}}_{\mathrm{PMm}}\left(0\right)=\frac{\sqrt{S_{\varphi m}\left(f_{x}\right)}}{|S_{\mathrm{PM}}\left(f_{x}\right)|}=\frac{f_{\mathrm{res}}\;\sqrt{2k_{B}T_{0}R_{m}}}{S_{\mathrm{mag}}{\hat{V}}_{\mathrm{ex}}Q}. \end{array}$$

**Figure 8:**
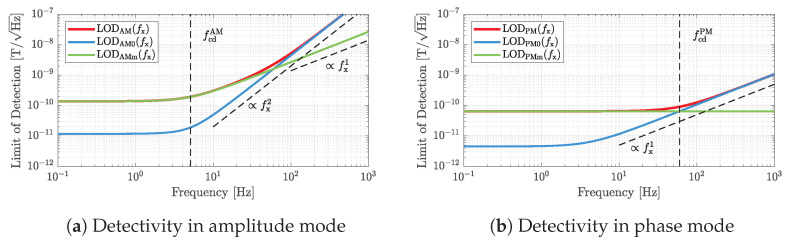
Illustrations of the limits of detection in AM mode (**a**) and in PM mode (**b**). For both types of operation, the low-frequency detectivity is limited by the thermal mechanical noise of the resonator. The bandwidth in which the detectivity is constant with frequency is significantly higher in PM mode. In general, the bandwidth can never be higher in AM mode than in PM mode. In addition, independent of the dominant type of noise, the detectivity in PM mode is always more than 6 dB better compared with an operation in AM mode. The plots are based on the sensor parameters given in Table 1.

Because both noise sources are additive noise (see above), the LOD can be improved in each case by increasing the excitation voltage ${\hat{V}}_{\mathrm{ex}}$. Figure 8 illustrates the detectivities for the exemplary magnetic field sensor considered in this article. For both operation modes, and in agreement with the previous noise analysis (see Figure 7), the low-frequency detectivities are limited by the thermal mechanical noise of the micromechanical resonator and are constant with the frequency. On the contrary, at higher frequencies and for both operating modes, the thermal electrical noise due to the piezoelectric layer limits the detectivity. While the detectivity in AM mode deteriorates with an increasing frequency proportional to $f_{x}^{2}$, the detectivity in PM mode increases only proportional to $f_{x}^{1}$ (compare the slopes of the associated dynamic sensitivities in Figure 5). In addition, the different dynamic sensitivities lead to significantly different cutoff frequencies at which the initially frequency-independent detectivities increase or deteriorate. These +3 dB cutoff frequencies can be considered as the bandwidths of the sensor system and are given by

(59) $$\begin{array}{c} f_{\mathrm{cd}}^{\mathrm{AM}}=\frac{f_{\mathrm{res}}\;\sqrt{2}\;\sqrt{\sqrt{R_{0}^{2}+8\;R_{0}R_{m}+10\;R_{m}^{2}}-R_{m}-R_{0}}}{4Q\;\sqrt{R_{m}}} \end{array}$$

in AM mode and by

(60) $$\begin{array}{c} f_{\mathrm{cd}}^{\mathrm{PM}}=\frac{f_{\mathrm{res}}\;\sqrt{R_{0}+R_{m}}}{2Q\;\sqrt{R_{m}}} \end{array}$$

in PM mode, respectively. It can be shown that $f_{\mathrm{cd}}^{\mathrm{AM}}\leq f_{\mathrm{cd}}^{\mathrm{PM}}$; thus, the bandwidth in terms of the limit of detection can never be higher in AM mode than in PM mode. With regard to the minimum achievable limit of detection inside the bandwidth, a distinction should be made between limitations by the different noise sources. If the detectivity is limited by the thermal electrical noise of the dielectric material, a low-frequency ratio of

(61) $$\begin{array}{c} \lim_{f_{x}\to 0}\frac{{\mathrm{LOD}}_{\mathrm{AM}0}\left(f_{x}\right)}{{\mathrm{LOD}}_{\mathrm{PM}0}\left(f_{x}\right)}=\frac{3\sqrt{3}\;\left(4\;Q-\sqrt{2}\right)}{8\;Q} \end{array}$$

is obtained. For practical Q>100 this relation approaches a value of $3\sqrt{3}/2\approx 2.5981$. Similarly, if the detectivity is limited by the thermal mechanical noise of the resonator, the ratio is given by

(62) $$\begin{array}{c} \lim_{f_{x}\to 0}\frac{{\mathrm{LOD}}_{\mathrm{AMm}}\left(f_{x}\right)}{{\mathrm{LOD}}_{\mathrm{PMm}}\left(f_{x}\right)}=\frac{3\;\sqrt{8\;Q^{2}-4\sqrt{2}\;Q+1}}{4\;Q} \end{array}$$

which, also for quality factors Q>100, approaches a value of $3\sqrt{2}/2\approx 2.1213$. Thus, independent of the dominant type of noise, the detectivity in PM mode is always more than 6 dB better compared with an operation in AM mode.

## 7. Conclusions

In this article, the performance of quasi-resonantly driven piezoelectric sensors operated in amplitude mode and phase mode has been investigated. Regardless of the physical quantity that a micromechanical sensor with a piezoelectric layer is designed to detect, changes in its resonance frequency result in both amplitude (AM) and phase modulation (PM) of the sensor signal if the sensor is operated in an open-loop configuration. The degree of the respective modulation is essentially determined by the frequency of the signal used to electrically excite the sensor.

The objective of the analysis presented here was to evaluate the respective performance in terms of the minimum reachable limit of detection (LOD) as a function of the frequency of the physical signal to be measured. Thus, based on the fundamental properties of micromechanical resonators, comprehensive analyses of the frequency-dependent sensitivities, as well as the frequency-dependent amplitude noise and phase noise, were performed and respective analytical expressions were derived. Different optimal excitation frequencies result for the two types of operation, and these also lead to different limits of detection. It has been shown that the low-frequency LOD in phase mode is always about 6 dB better than the LOD in amplitude mode. In addition, the bandwidth in terms of detectivity is generally significantly larger, and never worse, in phase mode compared with amplitude mode. Accordingly, an evaluation of the sensor signal’s phase with appropriate excitation of the sensor is generally preferable to an evaluation of the sensor signal’s amplitude.

To illustrate all the analytical relationships in this article, an exemplary piezoelectric magnetic field sensor was considered. In Table 2, all electromechanical parameters of this sensor, and further parameters of the employed sensor system, as well as various numerical values for the derived equations, are summarized.

Even if the exemplary sensor is a magnetic field sensor, all derived equations have general validity for all micromechanical sensors which can be described by the differential equation of motion of a damped driven harmonic oscillator (Equation (1)).

Several directions exist for the future continuation of the work presented here. First, confirmation of the theory developed here on the basis of measurements is advisable. Provided that the parasitic capacitance of the sensor is compensated as presented here, PM operation would always be preferred. However, it would also be interesting to compare AM and PM operation modes without neutralization, because the effective impedance leading to the electrical sensitivity can take a significantly different form. Due to the asymmetry caused by the additional parallel resonance, however, the calculations are considerably complicated, meaning that numerical simulation would possibly be more appropriate in this case.

## Figures and Tables

**Figure 7:**
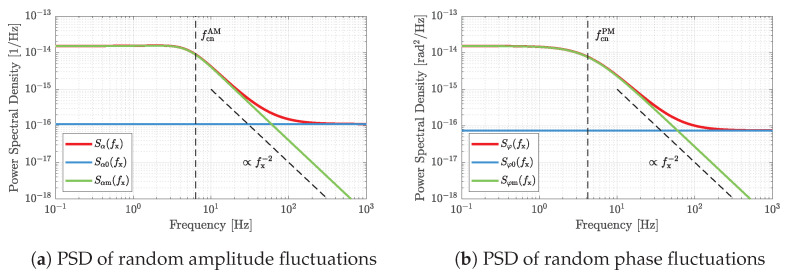
Illustrations of the power spectral densities (PSD) of random amplitude fluctuations (**a**) and random phase fluctuations (**b**). Both are composed of a constant component due to the thermal electrical noise of the dielectric and a frequency-dependent component due to the thermal mechanical noise. The plots are based on the sensor parameters summarized in Table 1.

**Table 1:** Electromechanical and magnetic properties of the exemplary magnetic field sensor referred to in this article.

Property	Value
Static capacitance	$C_{0}=44\;\mathrm{pF}$
Loss factor	$\tan\delta_{0}=5\cdot 10^{-3}$
Loss resistance	$R_{0}\left(f_{\mathrm{res}}\right)=97.5\;M\Omega$
Resonance frequency	$f_{\mathrm{res}}=7421.1\;\mathrm{Hz}$
Quality factor	Q=887.1
Motional resistance	$R_{m}=476.67\;k\Omega$
Motional capacitance	$C_{m}=50.71\;\mathrm{fF}$
Motional inductance	$L_{m}=9.07\;\mathrm{kH}$
Magnetic operating point	$B_{\mathrm{bias}}=0.65\;\mathrm{mT}$
Magnetic sensitivity	$S_{\mathrm{mag}}=80\;\mathrm{Hz}/\mathrm{mT}$

**Table 2:** Electromechanical properties of the exemplary magnetic field sensor referred to in this article and resulting numerical values for the derived equations.

Property	Value for AM	Value for PM
Static capacitance	$C_{0}=44\;\mathrm{pF}$	
Loss factor	$\tan\delta_{0}=5\cdot 10^{-3}$	
Loss resistance	$R_{0}\left(f_{\mathrm{res}}\right)=97.5\;M\Omega$	
Resonance frequency	$f_{\mathrm{res}}=7421.1\;\mathrm{Hz}$	
Quality factor	Q=887.1	
Motional resistance	$R_{m}=476.67\;k\Omega$	
Motional capacitance	$C_{m}=50.71\;\mathrm{fF}$	
Motional inductance	$L_{m}=9.07\;\mathrm{kH}$	
Magnetic operating point	$B_{\mathrm{bias}}=0.65\;\mathrm{mT}$	
Magnetic sensitivity	$S_{\mathrm{mag}}=80\;\mathrm{Hz}/\mathrm{mT}$	
Excitation amplitude	${\hat{V}}_{\mathrm{ex}}=1\;V$	
Feedback capacitance	$C_{f}=10\;\mathrm{pF}$	
Transimpedance	$|T(f_{\mathrm{res}})|=2.145\;M\Omega$	
Excitation frequency	$f_{\mathrm{ex}}^{\mathrm{AM}}=f_{\mathrm{res}}\mp 0.352\;\mathrm{Hz}$	$f_{\mathrm{ex}}^{\mathrm{PM}}=f_{\mathrm{res}}$
Electrical sensitivity	$S_{\mathrm{elec}}^{\mathrm{AM}}=\pm 112.7\cdot 10^{-3}\;1/\mathrm{Hz}$	$S_{\mathrm{elec}}^{\mathrm{PM}}=-239\cdot 10^{-3}\;\mathrm{rad}/\mathrm{Hz}$
Sensitivity cutoff frequency (−3 dB)	$f_{\mathrm{cs}}^{\mathrm{AM}}=4.35\;\mathrm{Hz}$	$f_{\mathrm{cs}}^{\mathrm{PM}}=4.18\;\mathrm{Hz}$
Overall sensitivity	$|S_{\mathrm{AM}}\left(1\mathrm{Hz}\right)|=8.9\;1/\mathrm{mT}$	$|S_{\mathrm{PM}}\left(1\mathrm{Hz}\right)|=18.6\;\mathrm{rad}/\mathrm{mT}$
Thermal electrical voltage noise density	$E_{d0}\left(f_{\mathrm{res}}\right)=27.5\;\mathrm{nV}/\sqrt{\mathrm{Hz}}$	
Thermal mechanical voltage noise density	$E_{\mathrm{dm}}\left(f_{\mathrm{res}}\right)=393\mathrm{nV}/\sqrt{\mathrm{Hz}}$	
Overall noise power spectral density	$S_{\alpha }\left(1\;\mathrm{Hz}\right)=1.55\cdot 10^{-14}\;1/\mathrm{Hz}$	$S_{\varphi }\left(1\;\mathrm{Hz}\right)=1.45\cdot 10^{-14}\;{\mathrm{rad}}^{2}/\mathrm{Hz}$
Noise cutoff frequency (−3 dB)	$f_{\mathrm{cn}}^{\mathrm{AM}}=7.0\;\mathrm{Hz}\neq f_{\mathrm{cs}}^{\mathrm{AM}}$	$f_{\mathrm{cn}}^{\mathrm{PM}}=4.18\;\mathrm{Hz}=f_{\mathrm{cs}}^{\mathrm{PM}}$
Overall detectivity	${\mathrm{LOD}}_{\mathrm{AM}}\left(1\mathrm{Hz}\right)=140.1\;\mathrm{pT}/\sqrt{\mathrm{Hz}}$	${\mathrm{LOD}}_{\mathrm{PM}}\left(1\mathrm{Hz}\right)=64.8\;\mathrm{pT}/\sqrt{\mathrm{Hz}}$
Overall detectivity cutoff frequency (+3 dB)	$f_{\mathrm{cd}}^{\mathrm{AM}}=5.1\;\mathrm{Hz}$	$f_{\mathrm{cd}}^{\mathrm{PM}}=60.0\;\mathrm{Hz}$

## Data Availability

Not applicable.
